# Household Slaveries

**Published:** 1860-09

**Authors:** 


### HALL'S JOURNAL OF HEALTH.

**Our Legitimate Scope is almost boundless: for whatever begets pleasurable and harmless feelings, promotes Health; and whatever induces disagreeable sensations, engenders Disease.**

WE AIM TO SHOW HOW DISEASE MAY BE AVOIDED, AND THAT IT IS BEST, WHEN SICKNESS COMES, TO TAKE NO MEDICINE WITHOUT CONSULTING A PHYSICIAN.

Vol. VII.] SEPTEMBER, 1860. [No. 9.

### HOUSEHOLD SLAVERIES.

BEING on the right side in reference to the "peculiar institution," and having been born and raised under its immediate influences, our opinions on that subject have been formed from observed facts and not from vain imaginations and false teachings. And now, after some years spent in these Northern climes, "far from home," we find it difficult to arrive at a satisfactory conclusion, which, under all the circumstances of the case, is the happiest and most cheerful class of persons,—the servants in a Southern kitchen, or the mistresses in a Northern parlor. Certainly a greater tyranny may not give as much unhappiness in ignorance, as petty annoyances may yield to culture and a higher civilization. And it may admit of debate, whether more smiles do not flit across the countenances of slavery in ordinary families, than illume the features of the daughters of the North in any twenty-four hours. Our Northern readers need not be at the trouble of imagining that we are about to defend slavery. We do not choose to do any thing of the sort, but introduce the subject for the purpose of wedging in a variety of practical truths in reference to human health and happiness, more especially connected with our domesticities.

In looking at the whole subject from a dispassionate standpoint, the most protrusive object in the mind's eye is the contrast between the mountain and the mouse; the grandeur and the ridiculousness; the magnificent myth of a free country in theory, and the contemptible narrow-mindedness of the reality, when the North unchurches, excommunicates and hands over to unending perdition all who speak for slavery; and the South imprisons, chains, tars and feathers, and hangs as high as Haman hung, all they can—catch! who speak against it. But we must say, that Southern folly and injustice carries away the "belt," in present parlance, in charging upon the whole North the follies and the crimes of a pitiful few; in accusing millions of doing what never entered the imagination of but some twenty persons to conceive. And now that the din and dust of last winter's commotions have passed away, let the South learn to act a wiser and a nobler part, in the event of the reöccurrence of contingencies, similar to those which all intelligent and good citizens, North and South, ardently hope may never be repeated. From opportunities of personal observation in the highways and byways of the North, which not many possess, we firmly believe that if any Southerner, from Governor Wise, up or down, could possibly be made to feel that an auger-hole would be a most welcome refuge, it would be the presentation of the actual facts of the case here in the North, in reference to the "John Brown Raid." The instinctive exclamation would bound forth: "What a fool I was, to get in such a pucker about nothing!" In a multitude of cases, in street or house, or car or steamer, it was impossible not to be struck with the general indifference to what the papers would make believe was the all-absorbing topic of the times. The facility with which it was dismissed, the careless levity with which it was handled, compelled the conviction, that the *great public* regarded it as a matter with which they had no special concern, as if they had said: "That is their business, not ours; let the South alone, to manage it in their own way."

SCENE I.—*City Cars.*

"What do you think of this John Brown affair?"

"Oh! stirring times these; makes things lively. What 's Erie to-day?"

SCENE II.—*Steamboat.*

"This slavery business seems to be looming up."

"Well! I don't know. Seems to me to be a bull movement of seedy politicians and hard-up newspapers, to raise a 'sell.' I bought a house at Yonkers the other day; it is the most delightful suburb of the city, accessible at almost any hour by rail or boat or horse; having a population, which for refinement and culture is perhaps not excelled. One of its sterling features, and which must commend it to the appreciation of all solid and conservative people, is the number and liberality of its churches; the care they take of their ministers, and the interest they show in all that pertains to good doing. But I am in a betweenity, locally and morally. I am equidistant from the Dutch Reformed and Episcopal churches, whose respective incumbents are the salt of the earth. Dominie Hulburt is one of the best of men. His heart is always in the right place (in his hand) ready to be given at a moment's notice, in kindly sympathy, in wise admonition, or cheerful counsel; a willing, patient and hard worker. My wife has Episcopalian predilections, and leans towards Mr. Carter, who will leave a mark for good behind him. All the carriages go there, and a great deal of money too; the latter returning never, except it may be in the way of bread cast upon the waters; the talented Rector contending that the ungiving, as well as the un-*for*-giving Christian, are pretty much in the same category."

SCENE III.—*Ferry-Boat.*

"I've seen a great deal in the papers about a rumpus in Virginia, but have not taken time to keep the run of it. Can you give me a bird's-eye view of the case before we get to the other side?"

"Certainly. Some old man, Smith, or Brown, or other short name, undertook to persuade some slaves to run off to the North, but they wouldn't do it; meanwhile the whites heard that something wrong was going on, surrounded the Brown people, killed some, took others prisoners, a few escaping. By the way, wouldn't you like to buy that lot of mine adjoining your house? will be sold at a great bargain to 'close a concern!'"

SCENE IV.—*Broadway.*

"Do you see that little thin man, walking briskly this way; shoulders stooping, eyes earthward, with slouched hat and walking-cane? That's Dr. Cheever."

"The man who has the 'wealth of —— anathema'?"

"The same."

"He ought to be hung!"

"Why?"

"The malignity which he exhibits against slaveholders, its aiders and abettors, is devilish."

"Hello—stop a minute, or you will show as much 'wealth' of malediction as he. Don't you believe in earnest men, consistent men?"

"Yes; consistency of faith and practice is a jewel, when exhibited in a good cause, whether in one of a class, a committee, or a country. If a man advocates temperance, or the sanctity of the Sabbath, and practices them, I care not how severely he comments upon the conduct of the enemies of both."

"Then you must be less severe in your rebuke of the occupant of the Puritan pulpit. He is an earnest man, impressive and fearless. No one in my hearing has ever spoke more eloquently and unsparingly against drunkenness and Sabbath-breaking; he has done great good thereby; and therein, as you must admit, deserves well of every Christian community."

"Well, I believe you are right, and for his fidelity and fearlessness, for his sincerity and his zeal in what he believes to be right, I am compelled to commend him. If he happens to be in the wrong, that's his own look-out. But the fact is, I believe it's none of my business any how. Things will rectify themselves. Let the South alone, to manage .ts own affairs. Why should I go into my neighbor's family on a formal call or social visit, and while receiving his confidence and courtesies, go into his kitchen, on a chance turning of his back, and persuade the occupants to leave him? By the way, where will you spend next summer? Newport is ruinously expensive. Saratoga is a human menagerie, the cages full of dust, all inclosed in a patent oven. The 'White Sulphur' is too far off. Beautiful Rockaway is too near for delightful exclusiveness. The older I get, the more I am convinced that the most comfortable spot for a summer's residence is New-York City, any where above Fourteenth street, between Third and Sixth Avenues. The difference between the range of a large house and that of a ten-by-twelve room, between a whole bed with a hair-mattress, and a bag of shucks across wooden slats, between the best and freshest fruits and berries which the country can afford, and the ring-streaked and speckled, which are retained as being 'good enough for boarders;' between milk drawn at midnight and placed fresh and cool on the breakfast-table, as by the Company in Tenth street, and that which is left after a journey to New-

York and back, under a broiling sun; between a hot roll or the light loaf of a baker, and the yellow saleratus biscuit or soggy home-made bread of a plowman's cook; between the morning walk on a clean foot-pavement with the animations of the street and the diversions of the shop-windows, and silent draggles through weeds, wet grass and dust, with nothing showing life but pigs and plowmen, the yelp of a dog, the squauk of a mud-puddle duck, the gabble of geese and the bellowing of cow-beaux; between the coolness of your own sheltered house at mid-day and a bald building on a barren hill without shade, broiling the whole day long under a summer's sun; between the short and quiet walk to church of a Sabbath morning, and the hurry-scurry and haste and bustle attendant on saddling horses and gearing carriages and other discomposing preparations for a journey of several miles and back—the difference, we say, between 'this and that,' is so wide in my judgment, that I have concluded to spend 'the season' at home, with the advantage that it costs nothing extra to do so. I will have neither to spend time, comfort, nor money; whereas, if I go to the Springs, every time I turn round or open my mouth, I must put my hand in my pocket. I must lose time from my business, be choked with dust in rail-cars, nauseated at the thought of hotel and steamboat cookery, nap on 'the rail' or dream on a shelf, while the clamor of baggage-men, the gauntlets of hack-drivers, and the imposition of porters are all standing and standard grievances hard to be borne."

Such conversations, to be heard any where and every where, showed to our eye that the great public sense of the North felt no special personal concern in a subject which at that very time was regarded by Southerners generally with such a deep and intense interest. We give it as our opinion, that the prevailing sentiment of Northerners, the bone and sinew of them, on the general subject of slavery is this, that in every present slave State, slavery and slaveholders shall be protected at any cost of blood and treasure which may be required of them for the same; that the Union will and shall be preserved; that whoever is elected President according to the law of the land, they will accept and obey, as they always have done; they are just as unalterable in their determination that domestic slavery as it now exists South of Mason's and Dixon's line, shall never be extended over one single additional inch of territory now belonging to the confederation, unless the majority of the people of the United States should vote for it. In such an event, they would most certainly acquiesce. Northern opinion on another subject is of interest. Millions of voters think that there is no necessity for meddling with slavery; that it is merely a matter of time, a matter of dollars and cents; that as soon as it becomes a non-paying institution it will cease to exist, and that it will as certainly become so, first on the borders, then inwards, as that the sun will shine to-morrow, and they believe themselves bound by solemn oath to bide that time. In this light, the unfairness and injustice of the South stood out in unenviable relief, when, for the fanaticism and murderous intent of twenty deluded men, they brought their accusations against the millions of Northerners besides, as generous and patriotic and as brave as themselves; they exhibited a want of magnanimity and confidence in their Northern brethren which is alien to the Southern heart. It is to be hoped that they will never again allow themselves to be misled by calculating politicians and hair-brained editors, both North and South, who merited the halter quite as much as the imbeciles, who, bent on robbery, violence and blood, if there was need for it, embarked their all and lost it, in an insane raid. Any meddling with slavery on slave soil, except to increase the safety of the master and to add to the comfort and happiness, present and future, of the slave himself, involves the triple crime of theft, perjury and murder, than which there can be no greater, no meaner, in all the black catalogue of human guilt. Such are the thoughts here.

"As we were saying," the upper crust of New-York, that is, ita leaders and rulers, or, to be more explicit, the sovereign —— wives of Gotham, for what is a husband nowadays? He is a mere machine to grind out money and hand it over to the powers that be, who know nothing as to how it comes and care less, so that it does come, and who spend it as freely as water for dress and show, for ribbons and feathers; if any is left, it is thrown into the "sinking fund" of the kitchen, which, if it hadn't a maw as large as a continent of greens, would have been filled to repletion long ago. And now we come to

*Firstly.* There is reason to believe that the amount of domestic discomfort in Northern homes, arising from the system of domestic help, is greater than in Southern households.

Speaking in general terms, the domestics of the North, the cooks, the chamber-maids, the nurses, the waiters and footmen, are foreign or native; the latter, whether black or white, are usually of no account, the colored dishonest, the whites above their business; as to the foreign, their business is above them, they are too insufferably stupid or too utterly careless and indifferent ever to learn; only two points engaging their attention, getting their wages and wasting as much as possible. About one in a hundred seems to have any moral sense of the duty of taking care of the interests of their employers. It is perfectly wonderful to witness sometimes the combination of insolence, pertness, and destitution of any sense of justice or of right, especially when is known the want and the degradation with which they were familiar in their own country—a year, a month! agone.

The foreign help is made up, mainly, of three classes, the Dutch, Scotch, and Irish. The Dutch are so dirty, we will let them alone; the Scotch drink whisky, and need not be counted on. The Irish are the main-stay of housekeepers, being too generally what phrenology terms "secretive," without moral principle, and seemingly incapable of taking any interest in the real welfare of the family which they are paid to serve. To squander, waste and destroy with utter recklessness, is the trait of how many we do not say; householders can name the "figure," which, in their own judgment, is just.

If they have been with you for years, they will leave you at a day's notice; if you discharge them short of a month's 'warning," they are "kilt entirely." If there is any time at which it gives them rather more pleasure to leave than any other, it is in the midst of preparation for company or of severe sickness in the family. The impudence of some of them is refreshing. A girl declined to engage herself because she observed that the front-gate was kept locked, "as if ye didn't want ainy one to come and say me at all at all." Another said, "I jis cum to say how yer house looked on the outside; it is too small entirely, and it's only brick; it don't suit, so I bid ye a good avening." A third came as chambermaid. "I like ye very much mam, indade. I see's ye're a lady, but I don't like having the little children to dress." A fourth was kept waiting, the mistress of the splendid mansion being necessarily detained, certainly not over ten minutes, when, on entering her own parlor, Biddy said: "Ye shouldn't kape me a waitin all this time." A very summary invitation into the street gave the young lady to understand that she had miscalculated. Another protested: "This is a very fine house and looks well kept, and no doubt ye're a fine lady and I would love to live wid the likes of ye, but the kitchen (twenty feet square) is too small."

They are extremely sensitive, too. A footman, at twenty dollars a month, left, because he was required to wash off the bricks in the back-area. Another, for being asked to wash the lower hall. "I'm not a maid of all-work." A neighbor got up—to no breakfast, because a servant being sick, there was a requirement to build a fire that morning. Another insisted on having tea for breakfast and dinner, as coffee gave her the head-ache. Another wasn't used to riddle out the coal, it looked mean. Very likely some of our city readers could give still livelier specimens of the ridiculous, but we have confined ourselves to what is literal and known.

It is a great indignity to require one to do what is the proper duty of another, whatever may be the emergency. "It's not my business, mam, to cut peraties." "I didn't hire to handle dirty clothes." Another came to "cook, and not to answer the door-bell when the waiter is out." The line of demarkation as to the particular duties of each, is to be defined with all the distinctness of a city survey or an insurance document, and to go beyond it is considered unendurable.

Many of them refuse to eat bread which has once been placed on their employer's table, although absolutely not touched, even with a fork; the result is, we have known a peck of sliced bread to be brought up at a time as the leavings of a week, and which may be seen any morning, to aid in filling the ash-barrel. We passed a charitable institution, in the ash-box of which had just been emptied a basket of good bread, in rolls and slices; reminding us of a bill brought in by the manager of an asylum in this city, about the time of the panic of 1857, for some fifty or more baskets of peaches, at three dollars a basket. Fine thing to be poor in New-York! or to be the money-handling officer of public charities! But this is off the subject; still, it is ominously suggestive of the sunnyside of poverty, and the reckless use sometimes made of charitable funds, by wasteful servants.

The first thing a new help does, when the employer is stupid enough to allow it, is to expatiate on the grand scale of living at her "last place." The lady "never comes into the kitchen. The gentleman never goes to market, but gives the money to the head-waiter, to make all the necessary purchases. I carried the keys all the time, mam, and when I left, the lady (and she was a fine lady too, mam) said she had never missed any thing while I was wid her, no indade and she said right. When we wanted tay, I wint to the chist and took the fill of my hand for one time; and we never used any thing but loaf-sugar, and it was well for me, for any other kind gives me the head-ache."

Not the least item is the rod which the cook in the kitchen holds over the mistress, who is so terribly afraid of being considered close and mean, that she dare not make any suggestion about burning less coal or gas, or serving up to a second meal any remnant of the preceding; so one thing goes on to another, until the mistress never gives a command, only makes a request; at length cookey becomes chief counselor of state, and reckless waste is the order of the day. The inevitable result of these things is, that many a freeman works harder and more incessantly than a slave, for he carries the labor of the brain to a restless bed, when the body-work of the day is over; still he does not "get along." The leaks in the kitchen are greater than the gains of the counter, and the red flag of the auctioneer closes the history.

In a Southern home, the wife is mistress of her own mansion; she expresses her wishes and they are quietly complied with; she retires to her chamber without any misgivings that she may have her own fires to build and her own breakfast to cook next morning; she has no apprehension that the hard earnings of her husband will be expended in filling stranger's mouths; if her servants help themselves ever so bountifully, she may congratulate herself that it is only a change of locality from shelf to stomach, that her "property" eats her property, and that the sum total is still her property, she can not be a loser, for she possesses just as much as she had before!

It is difficult to form an adequate conception of the amount of annoyance, irritation, fretfulness, and settled discomfort, which Northern wives suffer from unfaithful and incompetent domestics; it is the "skeleton in the house," the raw head and bloody bones of almost every family, Yet it must be confessed that a large share of it is due to the mistresses of New-York mansions. Many know literally nothing of kitchen or cookery, because nine out of ten of them were brought up with the implied understanding, or under the calculation, that they were to marry rich men, who could afford to have housekeepers. But where so many rich men were to come from, does not seem to have been a considered item in the matter. This is a fearful and an almost universal oversight on the part of managing mothers. Apparently, the "chief end," the absorbing and sole object of maid and mother, is to catch a husband and get a home; but how to make that home a happiness; how to throw around that husband such cords of love and affection, as will bind him to his own hearth, and make his home the eagerly sought retreat at each business day's ending, appears not to have been thought of; no preparation seems to have been made for it. By degrees, the husband finds, that for all the practical purposes of life, his wife is good for nothing. She can not work, can not sew, can not counsel, nor even sympathize with him, and in deep disgust or moody despair, he looks to the club-room, the gaming-table or the decanter for that solace, which he had a right to expect in the wife of his choice.

When the mistress knows nothing of domestic duties, it need not be surprising if the maid knows less. If the mistress does not take interest enough in her household to bend her mind to the learning of what would make her and her husband and children happier, there is no ground to complain that the servants do not care to learn.

The Irish are a sprightly race, and can take up a thing quickly, only if they have a teacher. But too often, the young wife knows nothing, can communicate nothing, and two ignoramuses are at the head of the household!

But there are other faults about young wives. They are passionate, hasty, inconsiderate. They know almost nothing about work themselves, and too often impose impossible amounts of labor on their servants; can make no allowance for the accidents of the kitchen; never think of giving words of encouragement; and as for sympathizing with a cook or laundress, that is out of the question; they think that their entire duty is performed in paying the wages agreed upon. How often those wages are kept back; how often reduced to the lowest possible amount; how often excessive washes are given out; how often it is twelve or one o'clock before the servants can retire, or other unreasonable things are done and required, may not be known; but they are matters of daily occurrence; and that minds uneducated should be gradually soured and grow selfish and indifferent, on account of these inconsiderations and injustices and unreasonable requisitions, is, after all, not very wonderful. If we would have better servants, we must ourselves be better; we must be more considerate, more courteous, and teach them, by having an interest in their welfare, how to take an interest in our own. Human life, in all its relations, is a series of generous reciprocities, and they who give them wisely, will receive them in return. We who are of higher cultivation must take the lead, in the exhibition of all good and noble qualities. The matron must lead the maid in patience and in forbearance; must encourage her to habits of industry, cleanliness, practicality and system in every department of the household; and by all means, be considerate; trying faithfully to do towards a domestic, as, if places were reversed, they themselves would like to be done to.

Our own experience in the way of Domestics in New-York has been fortunate. Our cooks have always been Irish or colored; we have seldom changed, and in each case on account of marriage or sickness; they have always been scrupulously neat, trustworthy and industrious, keeping good hours, with few or no visitors. There is something in physiognomy and general personal appearance; a great deal, in letting it be understood from the very first, that there must be prompt and implicit obedience; that every thing must be willingly done in precisely the way you want it, and in no other, without express permission. Keep your servants at a respectful distance; never hold colloquies with them; never allow a word to be repeated as to modes of management in other families; nor relations of what was said and done; of all mean things in creation, an itching ear for servants' tales is the meanest. If servants are treated considerately, kindly, patiently and courteously, many good qualities will be elicited thereby, and a gradual and certain elevation in tone and character, and general worth would be an inevitable result; and housekeepers owe it to themselves, and to one another, to promptly commence a reform on their own parts, with the assurance that good and encouraging results will immediately follow therefrom unto themselves; housekeeping will become less a care and more a pleasure; there will be fewer annoyances, fewer irregularities, more leisure, more quiet, more smiles and more health; and with these, home will be more inviting to the husband, the children will be less in the street; harmony, cheerfulness and good nature will bind together all hearts, and quiet, unity and comfort will pervade the household.

